# Blocking malaria transmission to *Anopheles* mosquitoes using artemisinin derivatives and primaquine: a systematic review and meta-analysis

**DOI:** 10.1186/1756-3305-6-278

**Published:** 2013-09-24

**Authors:** Solomon Mequanente Abay

**Affiliations:** 1 Department of Pharmacology, School of Medicine, Addis Ababa University, P.O. Box 9086, Addis Ababa, Ethiopia

**Keywords:** Malaria transmission blocking, Artesunate, Artemether-lumefantrine, Primaquine, Systematic review, Meta-analysis

## Abstract

**Background:**

Among the currently used drugs in malaria case management, artemisinin derivatives and primaquine have an impact on the transmissible stages of *Plasmodium falciparum*. Hence, they reduce the transmission of the parasite from the patient to the mosquitoes. The present study aimed to assess evidence for this hypothesis from controlled trials.

**Methods:**

All controlled clinical trials evaluating the transmission blocking activity of artemisinin derivatives and primaquine with or without other antimalarials were included in this systematic review. PubMed, Google Scholar, Web of Science, ScienceDirect, Medscape and the Cochrane library were systematically searched without language, publication status or date restrictions. The literature references were also scanned manually. The last search was run on July 15, 2013. Search terms included artemisinin derivatives, primaquine, malaria transmission, transmission blocking/reducing drugs and mosquito infection. The outcome measure was the mosquito infectivity rate after treatment of patients. Data were compared using odds ratio (OR), in random effects models.

**Results:**

Nine trials with a total of 13,831 mosquitoes were included in the meta-analysis. After combining the trials, the transmission of *P. falciparum* to *Anopheles* mosquitoes were lower in artesunate, artemether-lumefantrine and primaquine groups as compared with their control counterparts with OR of 0.36 (95% confidence interval (CI), 0.14-0.90), 0.49 (95% CI, 0.31-0.79) and 0.09 (95% CI, 0.01-0.73); respectively. In non-comparative longitudinal studies, the use of a single-dose of primaquine was shown to deter the transmission of malaria briefly.

**Conclusion:**

Evidence on the transmission blocking effect of artemisinin derivatives and primaquine is conclusive. Trials evaluating the combined impact of artemisinin derivatives and primaquine on malaria transmission is urgently needed.

## Background

Globally, an estimated 3.3 billion people were at risk of malaria in 2011, with populations living in sub-Saharan Africa having the highest risk of malaria infection [1]. Between 2000 and 2010, malaria mortality rates fell by 26% around the world. In the World Health Organization (WHO) African Region the decrease was 33%. During this period, an estimated 1.1 million malaria deaths were averted globally, primarily as a result of a scale-up of interventions with a proven track record [2,3]. However, malaria transmission still occurs in 99 countries around the world, and the malaria burden continues to cripple health systems in many African countries. In 2010, this entirely preventable and treatable disease caused an estimated 655,000 deaths worldwide [4].

Proven strategies for malaria control include early treatment of the illness with artemisinin-based combination therapies (ACTs) [5], intermittent preventive treatment for pregnant women (IPT_P_) [6], and using measures that reduce the risk of infection such as indoor residual spraying (IRS) or insecticide-treated nets (ITNs) [7]. These tools and strategies have shown to be effective at contributing to malaria control [8]. Among the drugs used currently in malaria case management, the ACTs and primaquine have an impact on the transmissible stages of *Plasmodium falciparum*. It is assumed that artemisinin derivatives act against young gametocytes, whereas primaquine acts on mature gametocytes, which are usually present in the circulation at the time when the patient presents for treatment [9].

Independent reports and a pooled data analysis study speculated about the malaria transmission reduction by artemisinin derivatives from malaria patients to *Anopheles gambiae*, a major malaria vector in Africa [10]. Similarly, many studies have reflected the impact of primaquine on malaria transmission from patients to *Anopheles stephensi*, a sub-tropical species that predominates in Asia. However, no systematic analysis has been performed on the transmission blocking activity of artemisinin derivatives and primaquine. The present study attempts to make a systematic review and meta-analysis of the impact of artemisinin derivatives and primaquine on malaria transmission to the vector, and to measure whether there are general trends across the different reports on the change in malaria transmission due to artemisinin derivatives and primaquine. The study also explores the reasons for variations across the reports.

## Methods

### Conduct of systematic review and search strategy

**Figure 1:**
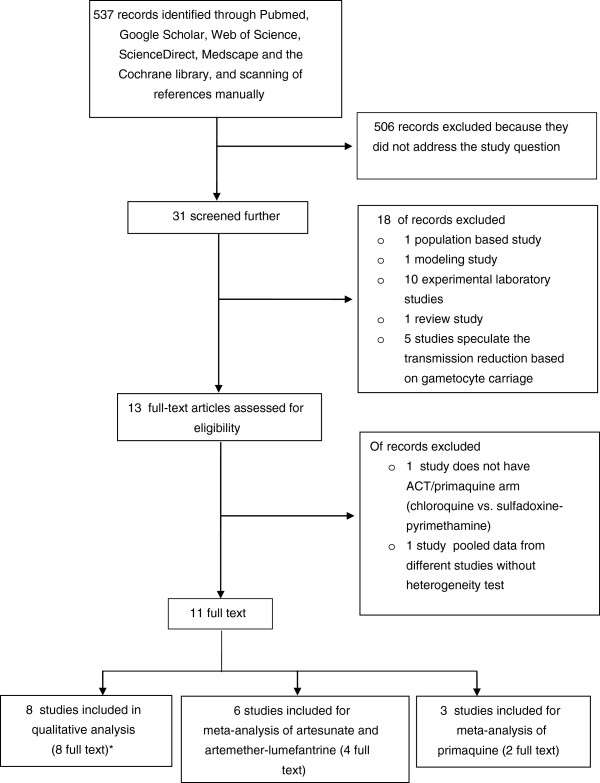
**Study selection flow diagram.** *three articles were used as sources of data for both qualitative and quantitative synthesis.

The investigator developed a protocol for this systematic review and conducted it in accordance with the PRISMA (Preferred Reporting Items for Systematic Review and Meta-Analyses) statement (Additional file 1: Checklist S1) [11]. A search was conducted to identify studies assessing malaria transmission blocking effect of drugs. PubMed, Google Scholar, Web of Science, ScienceDirect, Medscape and the Cochrane library were systematically searched without language, publication status or date restrictions. Literature references were also scanned manually. The last search was run on July 15, 2013. Search terms included artemisinin derivatives, primaquine, malaria transmission, transmission blocking/reducing drugs and mosquito infection (Additional file 2: Table S1). Studies comprising cases of *P. falciparum* treated with antimalarials (artemisinin derivatives and/or primaquine) and evaluating malaria transmission to *Anopheles* mosquitoes were included. Based on the inclusion criteria, studies for both artemisinin derivative (artesunate and artemether) and primaquine were selected for qualitative and quantitative synthesis. Figure 1 outlines the identification of studies for this systematic review and meta-analysis.

### Inclusion and exclusion criteria

Once it was determined that a paper contained a study on malaria transmission after interventions (artemisinin derivatives or primaquine), the full length article was consulted to check whether studies fulfill the following inclusion criteria for the meta-analysis:

 i. controlled clinical trial on malaria patients;

 ii. study on malaria transmission test from a patient to mosquito after treating the patient with the existing antimalarials; and

 iii. evaluating mosquito mid-gut for oocyst prevalence to measure the extent of malaria transmission.

Similar trials with the aforementioned criteria were also included for the qualitative synthesis. Studies involving laboratory animals or trials without control arms were excluded in the meta-analysis.

### Study design and outcome measures

The endpoints were mosquito infection prevalence out of all mosquitoes getting a blood-meal on patients after treatment with antimalarials. The endpoint evaluation was done in mosquitoes following their blood-meal directly on patients and artificial membrane feeding apparatus in primaquine trials and artemisinin derivatives; respectively. A data extraction sheet was used by the investigator to calculate the prevalence of mosquito infection in each trial. Information was extracted from each included trial on characteristics of trial participants, types of intervention (including type, dose, duration and frequency) and trial outcomes (mosquito infection prevalence). The odds of the end points from mosquitoes in the categories of artemisinin derivatives/primaquine and control (standard drugs) were calculated. The odds ratio (OR) and 95% confidence intervals (CI) for the prevalence of mosquito infection was then calculated.

The risk of bias in the studies used for meta-analysis was assessed by the investigator in an un-blinded manner using a tool developed by the Cochrane Collaboration (focusing on specific domains including random sequence generation, allocation concealment, blinding of participants and personnel, blinding of outcome assessment, incomplete outcome data, selective reporting) [12]. Due to the small number of studies identified, trials were not excluded based on quality assessment. For the same reason, publication bias was not assessed [12].

### Statistical analysis

Meta-analysis was performed using Meta-analyst, a software for meta-analysis of binary, continuous and diagnostic data [13]. The random effect method was used to test for differences in binary outcomes between artemisinin derivatives/primaquine and control (standard drug).

Heterogeneity, the variations among study outcomes, was checked by *tau*^2^ statistic, chi-square based Q-test, *I*^*2*^ statistics and heterogeneity P-value. The author chose the random effects meta-analytic model (DerSimonian and Laird) [14] to calculate the combined OR and 95% CI, even in a meta-analysis with low level of heterogeneity, in order to accommodate the random variation within the studies and the variation between the different studies.

## Results

### Description of included studies

The literature search identified the transmission blocking trials at the level of mosquitoes using two artemisinin derivatives (artesunate and artemether) and primaquine. Of 31 potentially relevant publications, only 6 publications (nine studies) on artemisinin derivatives and primaquine, fulfilled the inclusion criteria for the meta-analysis (Table 1). Eight publications were qualified for the qualitative analysis of transmission blocking property of primaquine and artemisinin derivatives.

**Table 1:** Characteristics of trials included in the meta-analysis

**Location** **(Study year)**	**Participants**	**Treatment arms**	**Mosquitoes (numbers used)**	**Mosquito infection method**	**Ref.**
Gambia* (2000)	Children (1–9 yrs)	Artesunate [a] + chloroquine [b]; chloroquine [b]	*Anopheles gambiae* (1208)	Membrane feeding	[15]
Gambia (1999)	Children	Artesunate [a] + sulfadoxine-pyrimethamine [f]; sulfadoxine-pyrimethamine [f]	*Anopheles gambiae* (3192)	Membrane feeding	[16]
Gambia (1998)	Children	Artesuante [g] + sulfadoxine-pyrimethamine [f]; sulfadoxine-pyrimethamine [f]	*Anopheles gambiae* (2688)	Membrane feeding	[16]
Kenya (2003–4)	Children (6 months–10 yrs)	Sulfadoxine-pyrimethamine [h] + placebo [i]; artesunate [a] + sulfadoxine-pyrimethamine [h]; sulfadoxine-pyrimethamine [h] + Amodiaquine [j]; artemether-lumefantrine [k]	*Anopheles gambiae* (3000)	Membrane feeding	[17]
Gambia (2002)	Children (1-10 yrs)	Artemether-lumefantrine [c]; chloroquine[d] + sulfadoxine-pyrimethamine [e]	*Anopheles gambiae* (540)	Membrane feeding	[18]
USA**	Adults	Primaquine [l]; Sulfadiazine + pyrimethamine [m]	*Anopheles stephensi* (30)	Direct skin bite	[19]
USA*** (1968)	Adults (25 to 41 yrs)	Primaquine [l]; pyrimethamine [n]	*Anopheles stephensi* (163)	Direct skin bite	[20]

a = 4 mg/kg for 3 days.

b = 25 mg/kg over 3 days: 10 mg, 10 mg, 5 mg.

c = Each dose: One half-tablet of co-artemether (20 mg artemether + 120 mg of lumefantrine) per 5 kg body weight up to 24 kg (two tablets).

d = Three daily doses of 10 mg/kg.

e = One half-tablet of SP (12.5 mg of pyrimethamine with 250 mg of sulfadoxine per half-tablet).

f = One-half tablet of pyrimethamine-sulfadoxine (12.5 mg pyrimethamine + 250 mg sulfadoxine) for a body weight of 10 kg & a further one-quarter tablet for each 5 kg increase.

g = 4 mg/kg immediate dose of artesunate.

h = 25 mg/kg sulfadoxine and 1.25 mg/kg pyrimethamine as a single dose plus.

i = placebo once daily for 3 days.

j = 10 mg/kg for once daily for 3 days.

k = administered as half a tablet (20 mg of artemether and 120 mg of lumefantrine) per 5 kg of body weight in a 6-dose.

l = single dose of 45 mg of primaquine base.

m = 500 mg of sulfadiazine every 6 hours for 5 days and 50 mg of pyrimethamine daily for 3 days.

n = Pyrimethamine 50 mg daily for 3 days.

*two parallel studies involving autologous plasma and control serum.

**the study year is not mentioned in the article.

***two parallel studies involving two strains of *P. falciparum*. Ref. = References.

Some of the publications, which fulfilled the inclusion criteria, had results of multiple studies; and these were used to generate effect sizes for the transmission blocking property of drugs (artesunate, artemether or primaquine) *vs.* controls. The studies used in the quantitative analysis were from Africa (Gambia and Kenya) and USA dealing with artemisinin derivatives and primaquine; respectively.

### Study quality assessment

A summary of the risk of bias assessment of the controlled trials can be found in Table 2. The extent of risk of bias varied across the studies. In general, trials done using artemisinin derivatives had a low risk of bias in most areas, whereas the trials on primaquine had an unclear risk of bias in most areas.

**Table 2:** Risk of bias assessment within the trials

**Study location (year)**	**Random sequence generation (selection bias)**	**Allocation concealment (selection bias)**	**Blinding of participants & personnel (performance bias)**	**Blinding of outcome assessment (detection bias)**	**Incomplete outcome data (attrition bias)**	**Selective reporting (reporting bias)**	**Other**	**Ref.**
Gambia (2000)	low	low	low	low	low	low	unclear	[15]
Gambia (1999)	low	unclear	low	low	low	low	unclear	[16]
Gambia (1998)	low	unclear	low	low	low	low	unclear	[16]
Kenya (2003–4)	low	unclear	low	low	low	low	unclear	[17]
Gambia (2002)	low	low	low	low	low	low	unclear	[18]
USA*	high	high	low	low	unclear	unclear	unclear	[19]
USA (1968)	high	high	low	low	unclear	unclear	unclear	[20]

*The study year is not mentioned in the publication. Ref = references.

### Heterogeneity assessment

Heterogeneity among the selected published studies was examined and the outcomes are presented in Table 3. A moderate to substantial heterogeneity was observed in the trials used for the two meta-analyses (with artesunate *vs.* without artesunate, primaquine *vs*. other antimalarials). The pooled ORs were calculated by the random-effects model, which assumes that the estimated effect size in different trials follow some distribution [21].

**Table 3:** Statistics to test heterogeneity in the meta-analysis

**Comparison**	**Heterogeneity**				Model used for meta-analysis*
	***Tau***^**2**^	**Q-value**	**P**_**heterogeneity**_	***I***^**2**^**(%)**	
With artesunate *vs*. without artesunate	1.18	79.7 (df = 5)	<0.01	94	Random
Artemether-lumefantrine *vs*. other antimalarials	0.05	2.77 (df = 2)	0.25	28	Random
Primaquine *vs*. other antimalarial	1.95	5.15 (df = 3)	0.16	42	Random

*For the reasons mentioned in the method (statistical analysis section), random effects model was chosen for all comparison.

### Artesunate-based combinations *versus* antimalarials without artesunate

**Figure 2:**
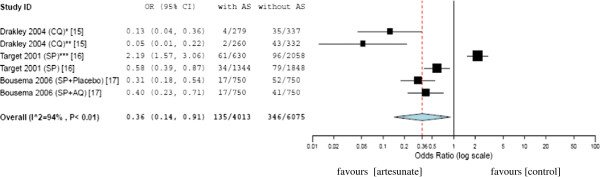
**Mosquito infectivity after feeding on blood from patients treated with antimalarials (with *vs. *without artesunate).** *control serum; **AP = autologous plasma; ***compared with single-dose artesunate; CQ = chloroquine; SP = sulfadoxine-pyrimethamine; AQ = amodiaquine; AS = artesunate.

The transmission blocking property of artesunate in combination with other antimalarials (chloroquine, sulfadoxine-pyrimethamine alone, sulfadoxine-pyrimethamine with amodiaquine or with placebo) were compared with the respective standard treatments (controls) without artesunate. The total number of mosquitoes checked for oocyst were 4,013 in the artesunate group and 6,075 in the control group. The OR was 0.36 (95% CI, 0.14-0.90), which favours artesunate-based combination therapy in reducing the transmission of *P. falciparum* parasite from patients to mosquitoes (Figure 2).

### Artemether-lumefantrine *versus* other antimalarials

**Figure 3:**
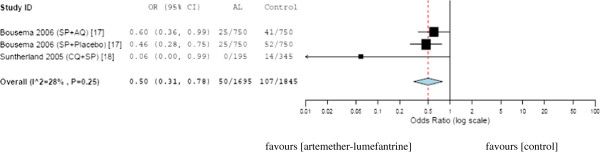
**Mosquito infectivity after feeding on blood from patients treated with artemether-lumefantrine versus control (standard therapy).** CQ = chloroquine; SP = sulfadoxine-pyrimethamine; AQ = amodiaquine; AL = artemether-lumefantrine.

The association between the use of artemether-lumefantrine and the reduction of malaria transmission was investigated. The total number of mosquitoes evaluated for oocyst was 1,695 in artemether-lumefantrine group and 1,845 in control group. The OR was 0.49 (95% CI, 0.31-0.79), which favours artemether-lumefantrine therapy in reducing the transmission of malaria from patients to mosquitoes (Figure 3).

The meta-analysis was in favour of artemisinin derivatives in reducing malaria transmission (Figures 2 and 3). In two randomised trials, the transmission blocking activity of ACTs were compared. In the first study, treatment with artemether-lumefantrine was associated with a lower proportion of infected mosquitoes as compared with dihyroartemisinin-piperaquine (OR 0.53; 95% CI, 0.37- 0.77) [22]. The second study comparing artemether-lumefantrine and artesunate in combination with sulfadoxine-pyrimethamine did not show any difference in the proportion of infected mosquitoes (OR 1.60; 95% CI, 0.87-2.98) [17].

### Primaquine single dose *versus* other antimalarials

**Figure 4:**
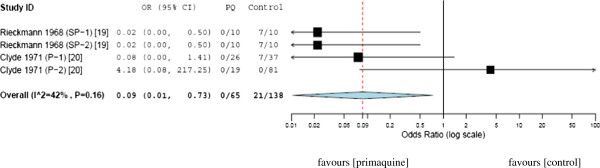
**Mosquito infection prevalence after blood-meal on primaquine treated malaria patients versus control (standard therapy).** PQ = primaquine; SP-1 and 2 = sulfadoxine-pyrimethamine in two independent patient group; P-1 and 2 = treatment in two patients infected with different strains of *P. falciparum*.

The association between single-dose primaquine (45 mg) and malaria transmission blocking from patients to mosquitoes was assessed. Overall, 65 mosquitoes in the primaquine group and 138 mosquitoes in the comparative group were used. The OR was 0.09 (95% CI, 0.01-0.73), which favours single-dose primaquine therapy in reducing the transmission of malaria from patients to mosquitoes (Figure 4).

### Single-dose of primaquine and malaria transmission at different points in time

**Figure 5:**
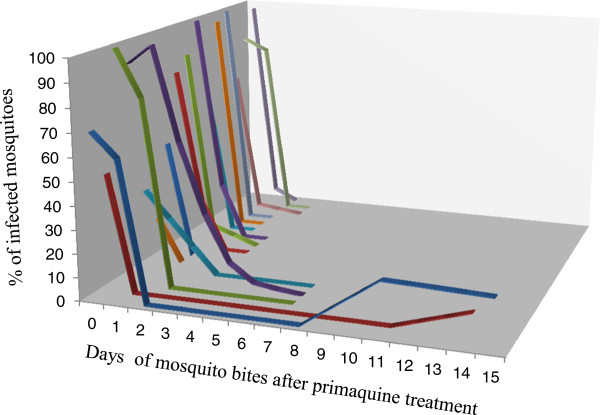
***Plasmodium *infectivity rate to mosquitoes that fed on falciparum malaria patients treated with a single dose of primaquine.** Day zero refers to the percent of oocyst positive mosquito fed on patients before the treatment. Sixteen of each line represents the oocyst positivity of mosquitoes feeding on single patients immediately before treatment and afterwards. Mosquitoes fed on two patients after 10 days of treatment were oocyst positive [19,20,23-25].

Single-dose primaquine from 15 mg to 45 mg were given to individuals diagnosed with *P. falciparum*, and the patients were exposed to mosquito bites at different days, i.e. before and after treatment. The data of mosquito infection rate *versus* time (treatment days) from 16 patients, reported by multiple studies, is presented in Figure 5.

In more than 93% of all the patients, the *Plasmodium* transmission to the mosquitoes had been blocked within the third day of administration of a single dose of primaquine. However, mosquitoes feeding on patients after 10 days of treatment became oocyst-positive. Briefly, this has been revealed in a study that followed patients for two weeks. Mosquitoes, fed on one patient on the 14th day and a second patient on the 11th and 15th days after primaquine treatment, became oocyst-positive (Figure 5).

### Addition of primaquine to antimalarials and malaria transmission: the mefloquine case

**Figure 6:**
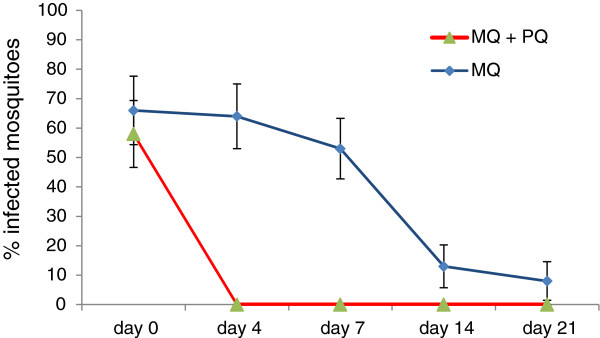
***Plasmodium *infective rate to mosquitoes after patients treated with mefloquine alone versus mefloquine plus primaquine.** The mosquitoes fed via membrane feeding on blood of patients treated with a single dose of mefloquine (750 mg) or a combination with primaquine single dose (45 mg). MQ = mefloquine; PQ = primaquine [26].

Chen and his colleague studied the impact of single-dose mefloquine with and without primaquine (45 mg) on the transmission rate of *Plasmodium* from patients to mosquitoes [26]. Before treatment, the mosquito infection prevalence was similar in mefloquine alone and mefloquine plus primaquine arms. Mosquitoes, fed on membrane feeding apparatus containing blood from patients treated with mefloquine plus primaquine on later days, were not infected unlike the group treated with mefloquine alone that showed a sluggish reduction in infection rate through time (Figure 6).

In the mefloquine plus primaquine group, the transmission of malaria was blocked throughout the duration of follow-up, i.e. 3 weeks (Figure 6). This duration of transmission blockade was longer than the duration (about 10 days) reported in those studies, which used only primaquine (Figure 5).

## Discussion

Preventing mosquitoes from biting by the existing tools (e.g. ITNs and IRS) and blocking the transmission of *Plasmodium* parasites from patients to mosquitoes using agents (vaccine or drugs) may substantially improve public health through reducing the burden of malaria [27]. In areas of stable malaria, a meta-analysis of ITNs on malaria transmission revealed a reduction by 50% [28]. However, a meta-analysis on existing antimalarials to block the transmission of the parasite from patients to mosquitoes has not yet been done to the author’s knowledge.

The current meta-analysis re-affirmed the advantage of ACTs in reducing transmission of *Plasmodium* to the mosquitoes. In the overall analysis, the odds of transmission of malaria in artesunate-based combination treatment group was 2.78 times less than the odds of transmission of malaria in antimalarials without artesunate (Figure 2). Similarly, the odds of malaria transmission to mosquitoes in artemether-lumefantrine treated group was 2 times less than the odds on other antimalarials used in the studies (Figure 3). Exceptionally, in one trial [16], the odds of parasite transmission was higher in artesunate-based combination therapy than the standard therapy without artesunate. This difference might be attributed to the use of only one dose of artesunate in the trial.

From the current analysis of the published articles, artesunate- and artemether-based therapy could reduce the transmission of the parasite to the mosquitoes. Thus, these medications, in addition to their role in the clinical case management of malaria, can have an impact on the overall transmission rate of malaria and play a role in controlling malaria.

Primaquine, the old antimalarial drug mainly used in terminal cure of *P. vivax* infection, was also tested for its impact on the transmission of *Plasmodium* to mosquitoes. Trials compared the transmission blocking effect of single-dose primaquine with the conventional antimalarials: sulfadoxine-pyrimethamine and pyrimethamine. The current analysis showed that odds of mosquito infection rate was 11 times less for single-dose primaquine relative to the comparative medicine (Figure 4).

In non-comparative longitudinal trials of single-dose primaquine, the current systematic review presented the trends of mosquito infection rate across successive post-treatment days (Figure 5). There was a rapid drop in mosquito infection rate as illustrated in those mosquitoes feeding on the first and second days post-treatment. However, the effect was maintained only for a short period as evidenced by the positivity of mosquitoes biting on some patients after the 10 days of single-dose primaquine therapy. As the trials employed single-dose primaquine therapy, which might target only the mature gametocyte [9], the continuity of transmission blockade by a single dose of primaquine fails against the new wave of mature gametocytes that appear after primaquine has been washed out [29].

The use of single-dose primaquine blocks the transmission of malaria temporarily (Figure 5). However, combining it with effective antimalarials - like mefloquine used in the trial conducted by Chen and his colleague - results in a disruption of *Plasmodium* transmission to the vector for a longer period (Figure 6). This might be explained by the fact that the effective schizonticide (mefloquine), by clearing the parasite, dries out the potential source for a new wave of gametocytes.

Following the introduction of ACTs in malaria case management, many researchers report the additional benefit of ACTs on the partial gametocytocidal effect compared with the non-ACTs regimens [30-34]. Recently, there is also interest of combining ACTs with single-dose primaquine, particularly in areas with a resistant *Plasmodium* to ACTs or in the phase of malaria elimination. The addition of primaquine to ACTs results in a further reduction in gametocyte carriage rate that can have implications on the extent of malaria transmission [35-39]. However, the optimal time of administration, treatment duration and dose of primaquine needs to be understood for improved outcomes. Further studies are also required to understand the transmission blocking effect upon the addition of primaquine to ACT regimens at the mosquito level, which is a gold standard approach to evaluate malaria transmission.

The present paper analysed the transmission blocking activity of artemisinin derivatives and primaquine. The clinical trials of artemisinin derivatives and primaquine focused on *An. gambiae* and *An. stephensi*; respectively. None of the identified literatures pertained to other dominant malaria vectors such as *An. funestus* and *An. arabiensis*[40-42]. In all of the trials included for meta-analysis, similar colonies of mosquitoes in both intervention and control groups were used. By so doing, confounding bias related to the variable infection success in the different species of vectors could be avoided. Of course, if the variability in susceptibility of the vectors to *P. falciparum* infection modified the transmission blocking activity of drugs, trials with different species of malaria vectors having a range of infection success would be required; then systematic reviews should consider subgroup analysis.

Like any retrospective study, this systematic review has a number of limitations. The literature search might have missed some trials. The author assumed that missing reports is unlikely for two reasons. Firstly, a proper search strategy was followed to identify literatures. Secondly, the research groups working on transmission blocking trials at the level of mosquitoes are few. Another concern is the comparison of the reference drugs (artemisinin derivatives and primaquine) with a heterogeneous array of antimalarials probably with different gametocytocidal activity, which in turn might obscure some systematic differences between drugs. To minimize these risk, random analytic method was followed in all data analysis, including in those with little heterogeneity.

## Conclusions

Based on the data reviewed, artemisinin derivatives (artesunate and artemether) and primaquine reduce the transmission of *P. falciparum* from the patient to the mosquitoes. Combining the two medications, after a proper optimization study, might further reduce the transmission of malaria.

## Competing interests

The author declares that he has no competing interests.

## Author contribution

SMA conceived the idea and designed the study, made both qualitative and quantitative synthesis of reported trials, wrote and revised the manuscript.

## Supplementary Material

Additional file 1 Checklist S1 PRISMA 2009 checklist. Click here for file

Additional file 2: Table S1 Search strategies for the databases. Click here for file

## References

[B1] World Health Organization Global Health Observatory http://www.who.int/gho/malaria/en

[B2] World Health Organization Malaria- factsheet on the world malaria report 2012 http://www.who.int/malaria/media/world_malaria_report_2012_facts/en/

[B3] Murray CJL Rosenfeld LC Lim SS Andrews KG Foreman KJ Haring D Fullman N Naghavi M Lozano R Lopez AD Global malaria mortality between 1980 and 2010: a systematic analysis Lancet 2012 379 413 31 22305225 10.1016/S0140-6736(12)60034-8

[B4] World Health Organization Media center- World malaria day http://www.who.int/mediacentre/news/releases/2012/malaria_20120424/en/

[B5] Price RN Nosten F Luxemburger C ter Kuile FO Paiphun L Chongsuphajaisiddhi T White NJ Effects of artemisinin derivatives on malaria transmissibility Lancet 1996 347 1654 8 8642959 10.1016/s0140-6736(96)91488-9

[B6] World Health Organization WHO Policy Brief for the Implementation of Intermittent Preventive Treatment of Malaria in Pregnancy using Sulfadoxine-Pyrimethamine (IPTp-SP) http://www.who.int/malaria/publications/atoz/Policy_brief_IPTp-SP_implementation_11april2013.pdf.pdf

[B7] Okumu FO Moore SJ Combining indoor residual spraying and insecticide-treated nets for malaria control in Africa: a review of possible outcomes and an outline of suggestions for the future Malar J 2011 10 208 21798053 10.1186/1475-2875-10-208 PMC3155911

[B8] Centers for Disease Control and Prevention Malaria: Current and Future Research http://www.cdc.gov/malaria/tools_for_tomorrow/research_areas.html

[B9] Wilairatana P Krudsood S Tangpukdee N Appropriate time for primaquine treatment to reduce *Plasmodium falciparum* transmission in hypoendemic areas Korean J Parasitol 2010 48 179 182 20585538 10.3347/kjp.2010.48.2.179 PMC2892577

[B10] Okell LC Drakeley CJ Ghani AC Bousema T Sutherland CJ Reduction of transmission from malaria patients by artemisinin combination therapies: a pooled analysis of six randomized trials Malar J 2008 7 125 18613962 10.1186/1475-2875-7-125 PMC2491628

[B11] Moher D, Liberati A, Tetzlaff J, Altman DG, The PRISMA Group Preferred reporting items for systematic reviews and meta-analyses: The PRISMA Statement PLoS Med 2009 6 e1000097 21603045 PMC3090117

[B12] Higgins JPT Green S Cochrane Handbook for Systematic Reviews of Interventions Version 5.1.0 http://www.cochrane-handbook.org

[B13] Wallace BC Schmid CH Lau J Trikalinos TA Meta-Analyst: software for meta-analysis of binary, continuous and diagnostic data BMC Med Res Methodol 2009 9 80 19961608 10.1186/1471-2288-9-80 PMC2795760

[B14] DerSimonian R Laird N Meta-analysis in clinical trials Control Clin Trials 1986 7 177 188 3802833 10.1016/0197-2456(86)90046-2

[B15] Drakeley CJ Jawara M Targett GAT Walraven G Obisike U Coleman R Pinder M Sutherland CJ Addition of artesunate to chloroquine for treatment of *Plasmodium falciparum* malaria in Gambian children causes a significant but short-lived reduction in infectiousness for mosquitoes Trop Med Int Health 2004 9 53 61 14728607 10.1046/j.1365-3156.2003.01169.x

[B16] Targett G Drakeley C Jawara M Seiden L Coleman R Deen J Pinder M Doherty T Sutherland C Walraven G Milligan P Artesunate reduces but does not prevent posttreatment transmission of *Plasmodium falciparum* to *Anopheles gambiae* J Infect Dis 2001 183 1254 9 11262208 10.1086/319689

[B17] Bousema JT Schneider P Gouagna LC Drakeley CJ Tostmann A Houben R Githure JI Ord R Sutherland CJ Omar SA Sauerwein RW Moderate effect of artemisinin-based combination therapy on transmission of *Plasmodium falciparum* J Infect Dis 2006 193 1151 9 16544256 10.1086/503051

[B18] Sutherland CJ Ord R Dunyo S Jawara M Drakeley CJ Alexander N Coleman R Pinder M Walraven G Targett GAT Reduction of malaria transmission to *Anopheles* mosquitoes with a six-dose of regimen o Co-artemether PLoS Med 2005 2 e92 15839740 10.1371/journal.pmed.0020092 PMC1087200

[B19] Rieckmann KH McNamara JV Frischer H Stockert TA Carson PE Powell RD **Gametocytocidal and sporontocidal effects of primaquine and of sulfadiazine with pyrimethamine in a chloroquine-resistant strain of*****Plasmodium****falciparum* Bull World Health Organ 1968 38 625 32 4876731 PMC2554527

[B20] Clyde DK Miller RM Music SI McCarthy VC Prophylactic and sporontocidal treatment of chloroquine-resistant *Plasmodium falciparum* from Vietnam Am J Trop Med Hyg 1971 20 1 5 4936022 10.4269/ajtmh.1971.20.1

[B21] Schrol JB Moustgaard R Gøtzsche PC Dealing with substantial heterogeneity in Cochrane reviews. Cross-sectional study BMC Med Res Methodol 2011 11 22 21349195 10.1186/1471-2288-11-22 PMC3056846

[B22] Sawa P Shekalaghe SA Drakeley CJ Sutherland CJ Mweresa CK Baidjoe AY Manjurano A Kavishe RA Beshir KB Yussuf RU Omar SA Hermsen CC Okell L Schallig HD Sauerwein RW Hallett RL Bousema T Malaria transmission after artemether-lumefantrine and dihydroartemisinin-piperaquine: a randomized trial J Infect Dis 2013 207 1637 45 23468056 10.1093/infdis/jit077

[B23] Clyde DK DuPont HI Miller RM McCarthy VC Prophylactic and sporontocidal treatment of chloroquine-resistant *Plasmodium falciparum* from Malaya Trans R Soc Trop Med Hyg 1970 64 834 838 4924648 10.1016/0035-9203(70)90102-1

[B24] Burgess RW Bray RS The Effect of a Single Dose of Primaquine on the Gametocytes, Garnetogony and Sporogony of *Laverania falciparum* Bull World Health Organ 1961 24 451 6 13689019 PMC2555915

[B25] Young MD The effect of small doses of primaquine upon malaria infections Indian J Malariol 1959 13 69 72

[B26] Chen PQ Li GQ Guo XB He KR Fu YX Fu LC Song YZ The infectivity of gametocytes of *Plasmodium falciparum* from patients treated with artemisinin Chin Med J 1994 107 709 11 7805466

[B27] Griffin JT Hollingsworth TD Okell LC Churcher TS White M Hinsley W Bousema T Drakeley CJ Ferguson NM Basáñez MG Ghani AC Reducing *Plasmodium falciparum* malaria transmission in Africa: a model-based evaluation of intervention strategies PLoS Med 2010 7 e1000324 20711482 10.1371/journal.pmed.1000324 PMC2919425

[B28] Lengeler C Insecticide-treated bed nets and curtains for preventing malaria Cochrane Database Syst Rev 2004 2 CD000363 10.1002/14651858.CD000363.pub2 15106149

[B29] Fletcher KA Evans DAP Gilies HM Greaves J Bunnag D Harinasuta T Studies on the pharmacokinetics of primaquine Bull World Health Organ 1981 59 407 12 6976848 PMC2396059

[B30] Carmona-Fonseca J Arango E Blair S Gametocytemia in falciparum malaria treated with amodiaquine or artesunate Biomedica 2008 28 195 212 18719722

[B31] Agomo PU Meremikwu MM Watila IM Omalu IJ Odey FA Oguche S Ezeiru VI Aina OO Efficacy, safety and tolerability of artesunate-mefloquine in the treatment of uncomplicated *Plasmodium falciparum* malaria in four geographic zones of Nigeria Malar J 2008 7 172 18782445 10.1186/1475-2875-7-172 PMC2542389

[B32] Zwang J Olliaro P Barennes H Bonnet M Brasseur P Bukirwa H Cohuet S D’Alessandro U Djimdé A Karema C Guthmann JP Hamour S Ndiaye JL Mårtensson A Rwagacondo C Sagara I Same-Ekobo A Sirima SB van den Broek I Yeka A Taylor WR Dorsey G Randrianarivelojosia M Efficacy of artesunate-amodiaquine for treating uncomplicated falciparum malaria in sub-Saharan Africa: a multi-centre analysis Malar J 2009 8 203 19698172 10.1186/1475-2875-8-203 PMC2745424

[B33] Sowunmi A Nkogho OO Okuboyejo TM Gbotosho GO Happi CT Adewoye EO Effects of mefloquine and artesunate mefloquine on the emergence, clearance and sex ratio of *Plasmodium falciparum* gametocytes in malarious children Malar J 2009 8 297 20015395 10.1186/1475-2875-8-297 PMC2805687

[B34] Osorio L Gonzalez I Olliaro P Taylor WR Artemisinin-based combination therapy for uncomplicated *Plasmodium falciparum* malaria in Colombia Malar J 2007 6 25 17328806 10.1186/1475-2875-6-25 PMC1820788

[B35] Pukrittayakamee S Chotivanich K Chantra A Clemens R Looareesuwan S White NJ Activities of artesunate primaquine against asexual and sexual stages parasites in falciparum malaria Antimicrob Agents Chemother 2004 48 1329 34 15047537 10.1128/AAC.48.4.1329-1334.2004 PMC375327

[B36] Smithuis F Kyaw MK Phe O Win T Aung PP Oo AP Naing AL Nyo MY Myint NZ Imwong M Ashley E Lee SJ White NJ Effectiveness of five artemisinin combination regimens with or without primaquine in uncomplicated falciparum malaria: an open-label randomised trial Lancet Infect Dis 2010 10 673 81 20832366 10.1016/S1473-3099(10)70187-0 PMC2947715

[B37] Eliana M Yulieth A Carmona-Fonseca J Efficacy of different primaquine-based antimalarial regimens against *Plasmodium falciparum* gametocytemia Acta Trop 2012 122 177 82 22245668 10.1016/j.actatropica.2012.01.005

[B38] Sutanto I Suprijanto S Kosasih A Dahlan MS Syafruddin D Kusriastuti R Hawley WA Lobo NF Ter Kuile FO The effect of primaquine on gametocyte development and clearance in the treatment of uncomplicated falciparum malaria with dihydroartemisinin-piperaquine in South Sumatra, Western Indonesia: an open-label, randomized, controlled trial Clin Infect Dis 2013 56 685 93 23175563 10.1093/cid/cis959

[B39] Shah NK Schapira A Juliano JJ Srivastava B Macdonald PD Poole C Anvikar A Meshnick SR Valecha N Mishra N A non-randomized controlled trial of artesunate plus sulfadoxine-pyrimethamine with or without primaquine for preventing the post-treatment circulation of *Plasmodium falciparum* gametocytes Antimicrob Agents Chemother 2013 57 2948 54 23587943 10.1128/AAC.00139-13 PMC3697316

[B40] Mwangangi JM Muturi EJ Muriu SM Nzovu J Midega JT Mbogo C The role of *Anopheles arabiensis* and *Anopheles coustani* in indoor and outdoor malaria transmission in Taveta District, Kenya Parasit Vectors 2013 6 114 23601146 10.1186/1756-3305-6-114 PMC3652741

[B41] Lwetoijera DW Kiware SS Mageni ZD Dongus S Harris C Devine GJ Majambere S A need for better housing to further reduce indoor malaria transmission in areas with high bed net coverage Parasit Vectors 2013 6 57 23497471 10.1186/1756-3305-6-57 PMC3599311

[B42] Tchouassi DP Quakyi IA Addison EA Bosompem KM Wilson MD Appawu MA Brown CA Boakye DA Characterization of malaria transmission by vector populations for improved interventions during the dry season in the Kpone-on-Sea area of coastal Ghana Parasit Vectors 2012 5 212 23013551 10.1186/1756-3305-5-212 PMC3495633

