# Supplementary material for: Blocking malaria transmission to Anopheles mosquitoes using artemisinin derivatives and primaquine: a systematic review and meta-analysis
Source: Parasit Vectors. 2013 Sep 24;6:278. doi: 10.1186/1756-3305-6-278 (PMC3849011; doi:10.1186/1756-3305-6-278)
Supplement: Additional file 2: Table S1 — Search strategies for the databases. [file 1756-3305-6-278-S2.docx]

**Table S1 Search strategies for the databases.** The same search strategy was used for all databases (Pubmed, Google Scholar, Web of Science, ScienceDirect, Medscape and the Cochrane library)

| **Search number** | **Search terms** |
| --- | --- |
| 1 | Artemisinin derivative |
| 2 | Artesunate |
| 3 | Artemether |
| 4 | Primaquine |
| 5 | Malaria transmission |
| 6 | Malaria transmission blocking drugs |
| 7 | Malaria Transmission reducing drugs |
| 8 | Mosquito infection |
| 9 | 1 & 5 or 6 or 7 or 8 |
| 10 | 2 & 5 or 6 or 7 or 8 |
| 11 | 3 & 5 or 6 or 7 or 8 |
| 12 | 4 & 5 or 6 or 7 or 8 |
